# ALIGNING VIDEO-AND STRUCTURED DATA FOR IMAGING OPTIMISATION

**DOI:** 10.1093/rpd/ncab071

**Published:** 2021-05-25

**Authors:** Jonas Ivarsson, Anja Almén, Mårten Falkenberg, Charlotta Lundh, Magnus Båth

**Affiliations:** Department of Applied IT, University of Göteborg, SE-412 96, Göteborg, Sweden; Department of Radiation Protection, Swedish Radiation Safety Authority, SE-171 16, Stockholm, Sweden; Medical Radiation Physics, Department of Translational Medicine (ITM), Lund University, SE-205 02, Malmö, Sweden; Department of Radiology, Institute of Clinical Sciences, Sahlgrenska Academy, University of Gothenburg, SE-413 45, Göteborg, Sweden; Department of Medical Physics and Biomedical Engineering, Sahlgrenska University Hospital, SE-413 45, Göteborg Sweden; Department of Radiation Physics, Institute of Clinical Sciences, Sahlgrenska Academy, University of Gothenburg, SE-413 45, Göteborg, Sweden; Department of Medical Physics and Biomedical Engineering, Sahlgrenska University Hospital, SE-413 45, Göteborg Sweden; Department of Radiation Physics, Institute of Clinical Sciences, Sahlgrenska Academy, University of Gothenburg, SE-413 45, Göteborg, Sweden

## Abstract

Imaging optimisation can benefit from combining structured data with qualitative data in the form of audio and video recordings. Since video is complex to work with, there is a need to find a workable solution that minimises the additional time investment. The purpose of the paper is to outline a general workflow that can begin to address this issue. What is described is a data management process comprising the three steps of collection, mining and contextualisation. This process offers a way to work systematically and at a large scale without succumbing to the context loss of statistical methods. The proposed workflow effectively combines the video and structured data to enable a new level of insights in the optimisation process.

Angiographic equipment for interventional procedures is used for a range of different treatments. However, the utilisation of these technologies is complex, and methods for optimisation must be considered for each procedure separately. Along with the emitted radiation, modern imaging equipment will also generate massive data streams about their settings and functioning. The collection, monitoring and assessment of this information are central to the optimisation process^(1–4)^. One challenge is that when the observed interventions are different from one another, variations in the structured data reports may be hard to interpret. Whereas one can discern the general trends, outliers and individual data points become impossible to fully understand because the unique conditions of these events are lost in the collection process. There is thus a need to develop a generally applicable strategy to overcome this issue.

The purpose of the paper is to outline a general workflow that can begin to address the difficulties with imaging optimisation by augmenting the stream of structured data with an additional source, that is, with qualitative data in the form of audio and video recordings. If these alternative sources can be combined intelligently and efficiently, it might offer a new prospect for the large-scale optimisation process of image-guided treatments. The separate steps of a workflow are described and exemplified with data from a research project. The focus for these materials will be on data processing matters, where the subsequent optimisation decisions and implementations are reported elsewhere^(5)^.

## VIDEO IN THE OPERATING ROOM

Many modern operating rooms are now equipped with recording instruments and ceiling-mounted cameras. The reasons for these installations may vary, but the sharing of knowledge is a recurrent argument. The very notions of the operating theatre, or the surgical amphitheatre, speak of a history where surgical suites were built with a dual purpose: the surgery itself and the teaching or performance in front of peers and students. With a developing understanding of asepsis, large live audiences not wearing scrubs were no longer invited to observe the events^(6)^. In this regard, video can nowadays displace the observer of a surgical procedure both in time and in space^(7)^. By recording surgeries, detailed analyses of the use of medical technologies^(8, 9)^ and specific work practices are enabled^(10, 11)^.

If such recordings are indeed collected, they may themselves serve multiple purposes. Technically, video uptakes should allow for the evaluation of imaging information in image-guided treatments to be also based on the physical and communicative work conducted. If analysed appropriately, these records may offer invaluable insights for imaging optimisation^(12)^. However, in practice, any systematic analysis of video-based materials is severely challenging and a very time-consuming process.

## A WORKFLOW FOR ALIGNMENT

The following section outlines a possible workflow for allowing the incorporation of video materials in the optimisation process. The proposed procedure follows the three steps of collection, mining and contextualisation.

### Step 1: collection

The first step leading up to the analysis is the collection and preparation of two separate datasets: structured data reports and video recordings, respectively. What is critical at this stage is to establish matching timelines for the separate datasets. In the simplest case, this only means that the video timestamps should be correctly set to enable synchronisation later. However, if multiple video-feeds are being collected, it might be necessary to carry out some more elaborate video-editing work to harmonise the separate streams.

Structured data reports (e.g. the Digital Imaging and Communications in Medicine (DICOM) standard) are standardised but may contain different parameters. Typically, they hold detailed information about the technique and dose parameters along with the precise time for each event. The suggestion is to convert this information into Time Series Data Frames in pandas, the Python Data Analysis Library (pandas.pydata.org), or something similar. Such a move enables subsequent manipulation and analysis.

### Step 2: mining

The second step constitutes the first part of the analysis. Here, only the structured data are examined and mined for potential insights. At this stage, a wide range of methods can be deployed. It is possible to work with everything from various machine learning techniques^(13)^ and statistical methods to mere visual inspections of different plots. The overarching purpose should be to identify regions of interest and to search for anomalies or systematic differences.

Such analyses may be sufficient in and of themselves. However, in a traditional approach, relying solely on structured data, the researcher would be barred from conducting in-depth analyses of many issues identified. The structured quantitative data tend to provide evidence that something occurred but not necessarily about why it happened on a specific occasion. The purpose of the proposed workflow is to disable this deadlock.

Given the additional video-based dataset and the aligned timelines, additional analytic avenues are opened. With this set-up, any identified occurrence in the structured dataset can be quickly located in the video materials and subjected to further scrutiny.

### Step 3: contextualisation

The final step is to make focused analyses of brief instances leading up to the identified occurrences of interest. Once such unique instances have been singled out from the structured data material, it is feasible to contextualise them with the help of the video. The circumstances surrounding the use of a specific protocol or the reasoning accompanying retaking an imaging sequence are now made available for analysis. By reviewing the choices made *in situ*, one can assess their significance concerning the larger picture. This root cause analysis provides valuable input to optimisation and can significantly advance our understanding of the situated use of different protocols and settings.

## ILLUSTRATIONS

### The data for the reported case

During 18 months, 70 procedures of endovascular aortic repair (EVAR) were carried out and documented. Out of these, 12 procedures were randomly selected to be additionally recorded on video. In total, 58 h were recorded with the aid of a ceiling-mounted camera in the operating room, a microphone near the operator and by capturing the operators’ screen. These separate video streams were combined into a single timeline using Final Cut Pro X (Apple Incorporated) software.

An Artis Zeego angiographic system (Siemens Healthineers) provided the structured data, such as, for instance, dose rates, time and the current settings.

### Digital subtraction angiographies protocols

**Figure 1:**
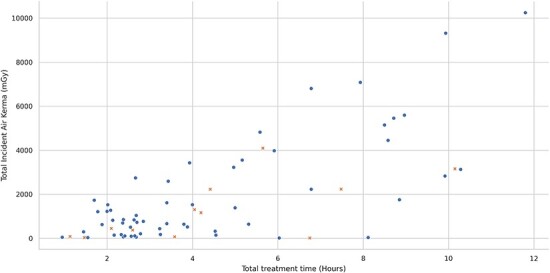
Scatter plot of the total dose (Incident Air Kerma) and duration for all treatments; the video-recorded treatments are marked with x.

The analysis started with a summary overview where it became evident that there were substantial differences in the levels of radiation used. The medical procedures varied in complexity, and the procedure time ranged between 1 and 12 h (see Figure 1).

However, these variations could not fully account for the differences in radiation levels between the procedures (see Table 1).

**Table 1:** The total dose (Incident Air Kerma) and duration for all treatments.

*N* = 70	Incident Air Kerma (mGy)	Duration (h)
Mean	1811	4.4
Standard deviation	2232	2.7
Min	13	1
First quartile	181	2.4
Median	842	3.4
Third quartile	2705	5.9
Max	10246	11.8

**Figure 2:**
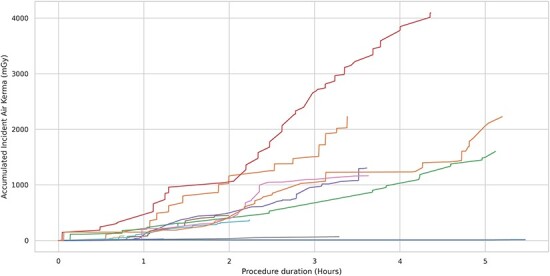
Roadmaps for all recorded treatments; each treatment is represented as a single line representing the accumulated Incident Air Kerma over time.

The treatments that had also been recorded were plotted as a function of the timewise accumulation of radiation. For each treatment, this would yield a unique pattern or roadmap (see Figure 2).

**Figure 3:**
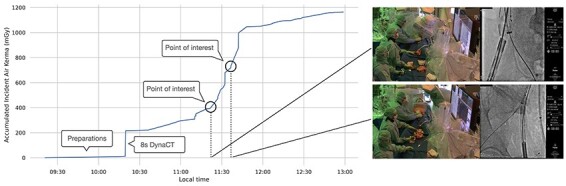
Roadmap with points of interest for one treatment; the marked regions indicate moments where the fluoroscopy dose rate is increased.

The stepwise increases evidenced most clearly in the procedures displaying the highest values were the results of digital subtraction angiographies (DSAs) and cone beam computed tomographies. While it is already known that DSAs generally come with a higher dose than fluoroscopy, the former were examined further.

The entire material contained 667 DSA acquisitions sequences that relied on one specific protocol in 47% of the cases. This half, however, accounted for 79% of the total Incident Air Kerma (in the reference point) produced during all these acquisitions. It thus became relevant to identify where and when this protocol could be replaced by one with lower settings.

Thus far, the examination had mainly worked from the structured data, but at this point, the analysis would need additional information. If different protocols and sensor settings should be recommended, those recommendations would also need to consider the differences in image quality that would be the result. In addition, the assessment of whether a single dose should be deemed too high or appropriate must build on the understanding of what problem the operator was trying to solve with that specific image acquisition. Hypothetically, for each case, conceivably, a higher dose was indeed needed to ground a diagnostic decision there and then. Alternatively, perchance the higher dose was avoidable.

These are precisely the kinds of questions that we can now begin to address. To advance to this next level of analysis, it becomes necessary to situate specific image acquisition sequences in the unique circumstances of their use. What were the problems encountered there, and what did it take to solve those? Based on this understanding, it then becomes possible to make recommendations for optimised use.

### Locating points of interest

A related example of how to work with contextualisation can be given by studying the roadmaps of individual treatments. The features of the plots can help guide the search for critical moments in the data.

As with the example of Figure 3, through visual inspection, it can be seen that the slope of the graph signifies the radiation dose rate. Three-dimensional CT and DSA appear as vertical lines, while the remaining sloping lines indicate different settings for fluoroscopy used by the operator. By studying this graph’s profile, it becomes possible to identify different phases of the procedure, and, within those phases, moments where the operator changes between settings can be located.

Guided by this search, points of interest can be identified—for instance, moments where there are significant changes in the profile of the graph. Having first identified these points, it becomes possible to interrogate the video materials and analyse the exact circumstances in which individual decisions were made.

With this method, it is suddenly possible to examine singular events that significantly affect the outcome in terms of radiation. These short episodes can be examined with video-based methods focussing on task-specific communication^(14–16)^. Concerning the example, the prevalence or absence of accounts motivating the change in dose would be relevant study objects. Also, it becomes possible to identify instances where some change in image acquisition settings was made but where the increase in image quality could not solve the actual problem encountered in that situation. By enabling the identification of such occurrences, the proposed workflow opens for new areas of improvements. In this way, the outlined approach can be considered as a form of situational optimisation.

## DISCUSSION

### Prerequisites and limitations

The proposed workflow comes with its prerequisites and limitations. First, the imaging equipment’s structured data reports should be systematically collected, which entails that relevant information is stored and made retrievable. Second, additional obstacles are found with the production and management of video recordings. At hospitals where cameras are present, it can be difficult to save those recordings to disc. Furthermore, even with such systems in place, there may be a lack of infrastructural support for large video materials’ long-term storage.

## CONCLUSION

The described situational optimisation can draw on the rich and contextual information that video records afford while simultaneously avoiding some of the considerable drawbacks with this method. Video materials are complex, and any work on them is time-consuming. The outlined approach, however, can significantly reduce the time spent on convoluted materials. It offers a way to work systematically and at a large scale without succumbing to the context loss of statistical methods. The proposed workflow could thereby enable a new level of insights to inform and guide the optimisation process.
